# The β-glucan from *Lentinus edodes* suppresses cell proliferation and promotes apoptosis in estrogen receptor positive breast cancers

**DOI:** 10.18632/oncotarget.21411

**Published:** 2017-09-30

**Authors:** Hui Xu, Siwei Zou, Xiaojuan Xu

**Affiliations:** ^1^ College of Chemistry and Molecular Sciences, Wuhan University, Wuhan 430072, China

**Keywords:** β-glucan, breast cancer, estrogen receptor positive, proliferation, apoptosis

## Abstract

Breast cancer is now the most common cancer in worldwide women, and novel interventions are needed to overcome the resistance occurring in the estrogen-targeted endocrine therapy. Herein, we demonstrate that the β-glucan from *Lentinus edodes* (LNT) exhibited a profound inhibition ratio of ∼53% against estrogen receptor positive (ER+) MCF-7 tumor growth in nude mice similar to the positive control of cisplatin. Immunohistochemistry images showed that LNT evidently suppressed cell proliferation and promoted apoptosis in MCF-7 tumor tissues. The Western blotting analysis indicated that LNT up-regulated the tumor suppressor p53, phosphorylated extracellular signal-regulated kinase1/2 (p-ERK1/2), cleaved-Caspase 3 and poly [ADP (ribose)] polymerase 1 (PARP 1) protein levels, and reduced the expression of mouse double minute 2 (MDM2), telomerase reverse transcriptase (TERT), nuclear factor-kappa B (NF-κB) p65, B-cell lymphoma-2 (Bcl-2), estrogen receptor α (ERα), etc. in tumor tissues. Moreover, LNT significantly suppressed phosphatidylinositol 3-kinase (PI3K), phosphorylated protein kinase B (p-Akt) and mammalian target of rapamycin (mTOR) protein levels. It was thus proposed that LNT inhibited MCF-7 tumor growth through suppressing cell proliferation and enhancing apoptosis possibly via multiple pathways such as PI3K/Akt/mTOR, NF-κB-, ERK-, ERα-, caspase- and p53-dependent pathways. Interestingly, the cell viability assay, siRNA transfection, Western blotting and flow cytometric analysis suggested that LNT targeted p53/ERα to only suppress cell proliferation via cell cycle arrest at G2/M phase without apoptosis *in vitro*. The big difference between *in vivo* and *in vitro* data suggested that the immune responses triggered by the polysaccharide should mainly contribute to the apoptotic effect *in vivo*. Overall, this work provides a novel strategy to treat ER+ breast cancers by using a naturally occurring β-glucan from mushrooms.

## INTRODUCTION

Breast cancer is the most commonly diagnosed and the leading cause of cancer-related death in women worldwide [1]. According to GLOBOCAN statistical analysis in 2012, 1.67 million women were diagnosed with breast cancer worldwide, accounting for 25.2% of all cancers among women [2]. With constantly increasing incidences of female breast cancer [3], more resources should be invested in primary prevention, earlier diagnosis and better health services to increase survival rates among global females. Approximate 70-75% of breast cancers express the estrogen receptor α (ERα), which are considered ER positive (ER+) [4]. That is, most breast tumor growth is dependent on estrogen. Therefore, endocrine therapy becomes the first class of target-directed therapy approved for treatment of breast cancer. However, only about 20-40% clinical patients with advanced ER+ breast cancer derive benefit from endocrine therapy due to the acquired resistance [4]. Therefore, novel anticancer interventions are needed to overcome the resistance and increase the therapeutic response while minimizing systemic side effects. A promising approach has been proposed to develop more effective, nonendocrine, and nontoxic therapeutic strategies using natural products owing to their cancer preventive and therapeutic potential [5, 6]. Particularly, Youyou Tu in China was awarded the 2015 Nobel Prize for physiology and medicine owing to the discovery of natural artemisinin as a drug to save millions of lives with malaria across the globe [7]. Therefore, discovery and development of anticancer drugs from natural resources will become one of the mainstreams of drug discovery in the near future due to the abundant natural resources in the land and ocean.

Lentinan is a β-(1→6) branched β-(1→3)-glucan derived from the mushroom of *Lentinus edodes* [8], which has been licensed as the drug for gastric cancer treatment in Japan [9]. The clinical studies have shown that chemo-immunotherapy using Lentinan prolongs the survival of patients with advanced gastric cancer compared with chemotherapy alone [9]. So far, there are six Lentinan injections or powders for injection used clinically in China [10]. Since 1970s, extensive studies have shown Lentinan alone or in combination with other chemotherapeutic drugs can be used for treating ovarian cancer [11], gastric cancer [9], hepatic carcinoma [12], and lung cancer [13]. However, the anticancer mechanism in all the tumors or cancers is far from conclusive. The recent work demonstrated that Lentinan activated immune responses to induce cell apoptosis and to suppress cell proliferation via caspase 3- and p53-dependent signaling pathways, leading to Sarcoma 180 tumor growth inhibition [14]. In viewing the literatures, very few reports on Lentinan against breast cancers are found [15]. Therefore, in this study, we focused on the anticancer effect of Lentinan (LNT for short) against breast cancers and the possible mechanism by using confocal microscopy, Western blotting, histology and immunohistochemistry, immunofluorescence, flow cytometry, etc. Consequently, LNT showed remarkable anti-proliferation effect against ER+ breast cancer cells *in vitro* and in nude mice. Moreover, LNT promoted cell apoptosis possibly via multiple pathways, contributing to ER+ breast tumor growth inhibition *in vivo*. This work provides an alternative strategy to treat ER+ breast cancers by using a naturally occurring β-glucan from mushrooms, as well as a deep insight into the anticancer mechanism of β-glucans.

## RESULTS

### LNT selectively inhibits viability/proliferation of ER+ breast cancer cells via cell cycle arrest *in vitro*

**Figure 1:**
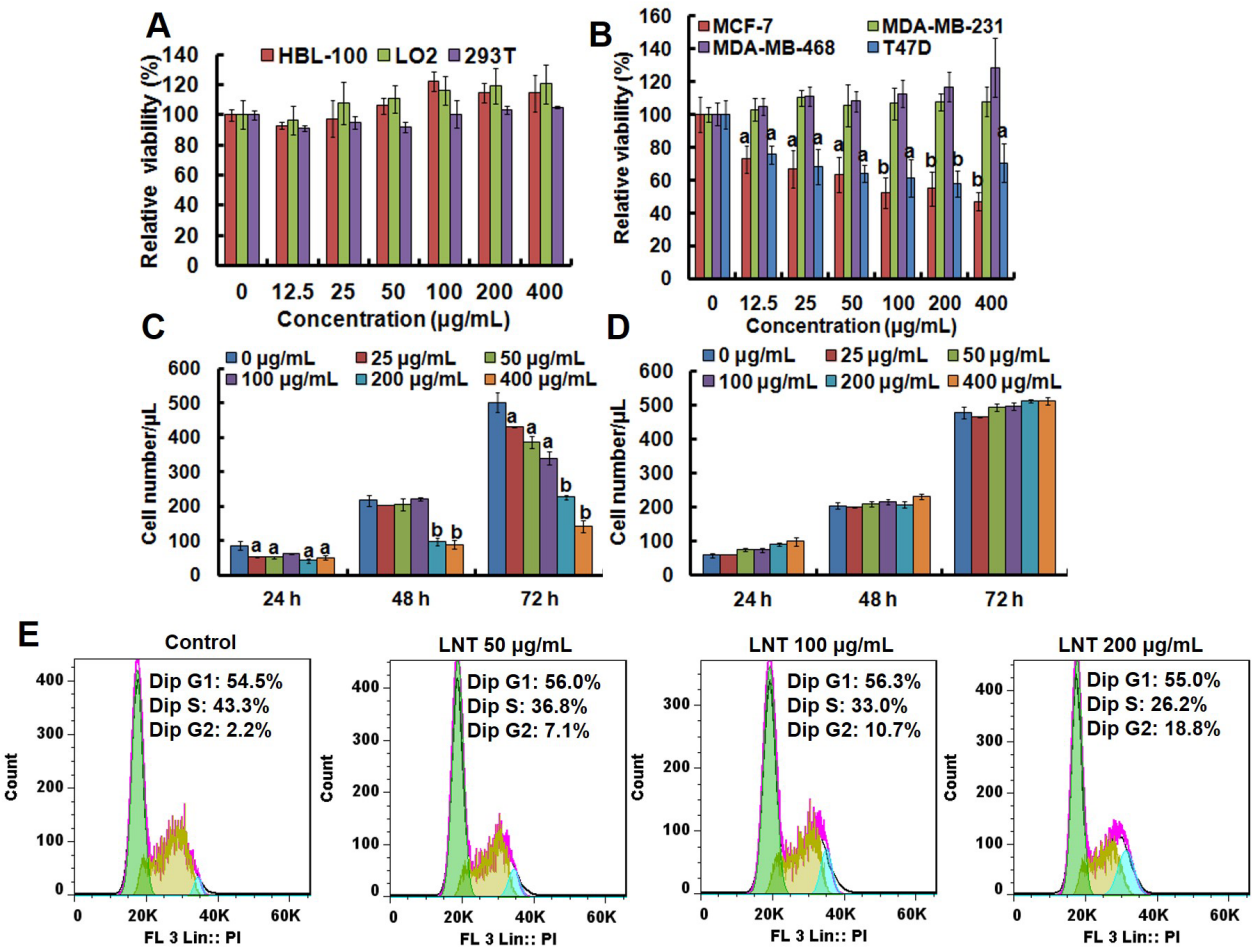
Anti-tumor effects of LNT and cell cycle analysis in MCF-7 cells *in vitro* **(A)** Cell viability of human normal cells of HBL-100, LO2 and 293T after treatment with LNT for 48 h determined by MTT assay. **(B)** Cell viability of different human breast cancer cells after treatment with LNT for 72 h determined by MTT assay. **(C, D)** Cell proliferation of MCF-7 and HBL-100 cells measured by trypan blue dye-exclusion assay, respectively. ^a^*p* < 0.05 and ^b^*p* <0.001 versus the control (PBS) at the respective incubation time point. **(E)** Cell cycle arrest induced by LNT. MCF-7 cells were incubated with LNT at 0, 50, 100 and 200 μg/mL for 24 h, and cell cycle distribution was determined by using the flow cytometry.

In this study, four human breast cancer cell lines of MCF-7, T47D, MDA-MB-231 and MDA-MB-468 were selected to evaluate the cytotoxicity of LNT. As shown in Figure 1A, LNT had not visible effect on the cell viability of human normal cells including breast cell (HBL-100), hepatocyte cell (LO2) and embryonic kidney cell (293T), but evidently repressed MCF-7 and T47D cell viabilities in a dose-dependent manner (Figure 1B and Supplementary Figure 1). Interestingly, LNT treatment hardly caused growth inhibition in MDA-MB-231 and MDA-MB-468 cells (Figure 1B and Supplementary Figure 1). As known, MCF-7 and T47D cells are estrogen receptor positive (ER+), while MDA-MB-231 and MDA-MB-468 cells are estrogen receptor negative (ER-) [16]. The results indicated the cell-type specific cytotoxicity of LNT and a good safety profile in normal cells. To observe whether LNT induced cell death, trypan blue dye-exclusion assay was performed. As a result, LNT effectively reduced the number of MCF-7 cells in both dosage- and time-dependent manners (Figure 1C), but did not affect the normal breast cell number of HBL-100 at all concentrations used (Figure 1D). Importantly, blue dead cells stained by the trypan blue dye were not observed in MCF-7 cells, suggesting that LNT mainly caused MCF-7 cell proliferation inhibition *in vitro* and did not directly induce tumor cell apoptosis or death.

As well known, cell cycle arrest plays an important role in the inhibition of proliferation [17]. And cell cycle phase distribution of MCF-7 cells after LNT treatment for 24 h was measured by flow cytometry. As shown in Figure 1E, with increasing LNT concentrations, the percentage of MCF-7 cells at G2/M phrase significantly increased from 2.2 to 18.8%, indicating that LNT predominantly induced G2/M phase cell cycle arrest in a dose-dependent manner for preventing cancer cells from division, contributing to the proliferation inhibition in Figure 1C. In accordance with the trypan blue dye-exclusion assay result, Sub-G1 phase standing for apoptotic cells was not detectable. These findings suggested that LNT specifically suppressed proliferation of ER+ breast cancer cells as a major contribution to cell growth inhibition via cell cycle arrest *in vitro*.

### Internalization of LNT by MCF-7 cells

**Figure 2:**
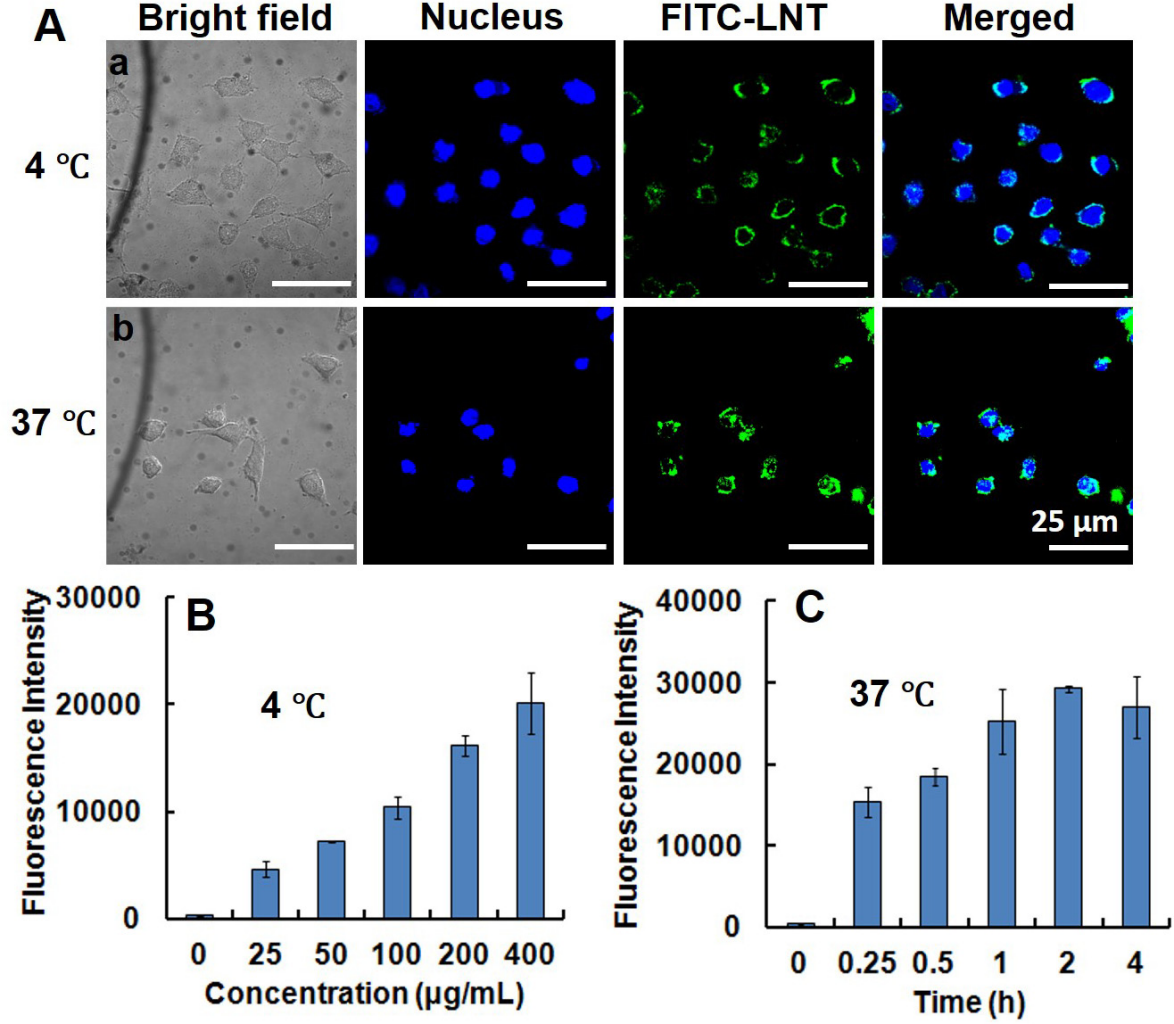
Interaction of LNT with MCF-7 cells *in vitro* **(A)** Recognition and internalization of LNT by MCF-7 cells. After incubation at 4°C for 40 min (a) and at 37°C for 2 h (b) with FITC-LNT (200 μg/mL), MCF-7 cells were stained with Hoechst 33342, fixed and imaged by confocal microscopy using laser excitation at 405 and 488 nm. The micrographs were obtained at a magnification of 600 ×. **(B, C)** Cellular uptake of FITC-LNT quantitatively estimated by the fluorescence intensity of cell lysates treated with different concentrations at 4°C for 40 min and with FITC-LNT (200 μg/mL) at 37°C for different times. FITC-LNT was used as the reference. Values expressed are means ± standard deviation (SD) of triplicates. Scale bars, 25 μm.

It has been reported that β-glucans from yeast, fungi, grain and seaweed adopt the pathogen associated molecular pattern (PAMP), so that they can trigger specific recognition and internalization by some cells [18]. To clarify whether LNT was recognized or internalized by MCF-7 cells, LNT was conjugated with FITC (denoted FITC-LNT) and incubated with MCF-7 cells at 4°C, followed by a confocal microscopy observation. Consequently, the circular green color standing for FITC-LNT was observed to bind to the surface of MCF-7 cells (Figure 2A), indicative of recognition of LNT by MCF-7 cells [19–21]. To confirm the attachment of LNT to the surface of MCF-7 cells, FITC alone was used as the control. As shown in Supplementary Figure 2, many green colors of FITC entered into MCF-7 cells due to small size of FITC and almost overlapped with the blue-stained nuclei even at 4°C. This image was completely different from FITC-LNT. These findings powerfully demonstrate that it was LNT but not FITC bound to the surface of MCF-7 cells in Figure 2A. At 37°C, except the circular green color, some green colors overlapped with the blue nuclei (Figure 2A), suggesting some FITC-LNT samples were internalized into cells. The fluorescence intensity of FITC-LNT in MCF-7 cell lysates was quantified by colorimetry. It is worth noting that the internalization behavior of macromolecules can be inhibited without affecting the binding ability at low temperature [22]. Therefore, the fluorescence intensity was completely ascribed to the binding of FITC-LNT to MCF-7 cell surface at 4°C. As shown in Figure 2B, the fluorescence intensity increased with increasing the FITC-LNT concentration at 4°C, suggesting that more FITC-LNT molecules bound to MCF-7 cells at the elevated concentration of LNT. At 37°C, the fluorescence intensity was higher than that at 4°C, which increased with increasing the incubation time of LNT with MCF-7 cells and reached the maximum at 2 h (Figure 2C). It can be concluded that LNT was really internalized by MCF-7 cancer cells.

As well known, β-glucan binds to receptors such as dectin-1 and complement receptor 3 (CR3, CD11b/CD18) [23] on the surface of immune cells for initiating the immune responses. To examine whether these receptors express on the surface of MCF-7 cancer cells, confocal microscopy observation was performed. As shown in Supplementary Figure 3, it is surprising that dectin-1 and the subunit CD11b of CR3 were clearly observed. It triggered us to study if LNT inhibited MCF-7 cell proliferation through dectin-1 or CR3. After blocking dectin-1 and CR3 on the surface of MCF-7 cells, the cell viability inhibition by LNT was not affected (data not shown here). These findings tentatively suggested that dectin-1- or CR3-dependent pathways were possibly not involved in the cell proliferation inhibition by LNT. It is possible that LNT bound to some other unknown receptors on the MCF-7 cell surface to trigger the cell proliferation *in vitro*; herein the exact binding of LNT to MCF-7 cell surface remains unclear, and further intensive studies are needed.

### LNT targets p53/ERα to inhibit MCF-7 cell proliferation *in vitro*

**Figure 3:**
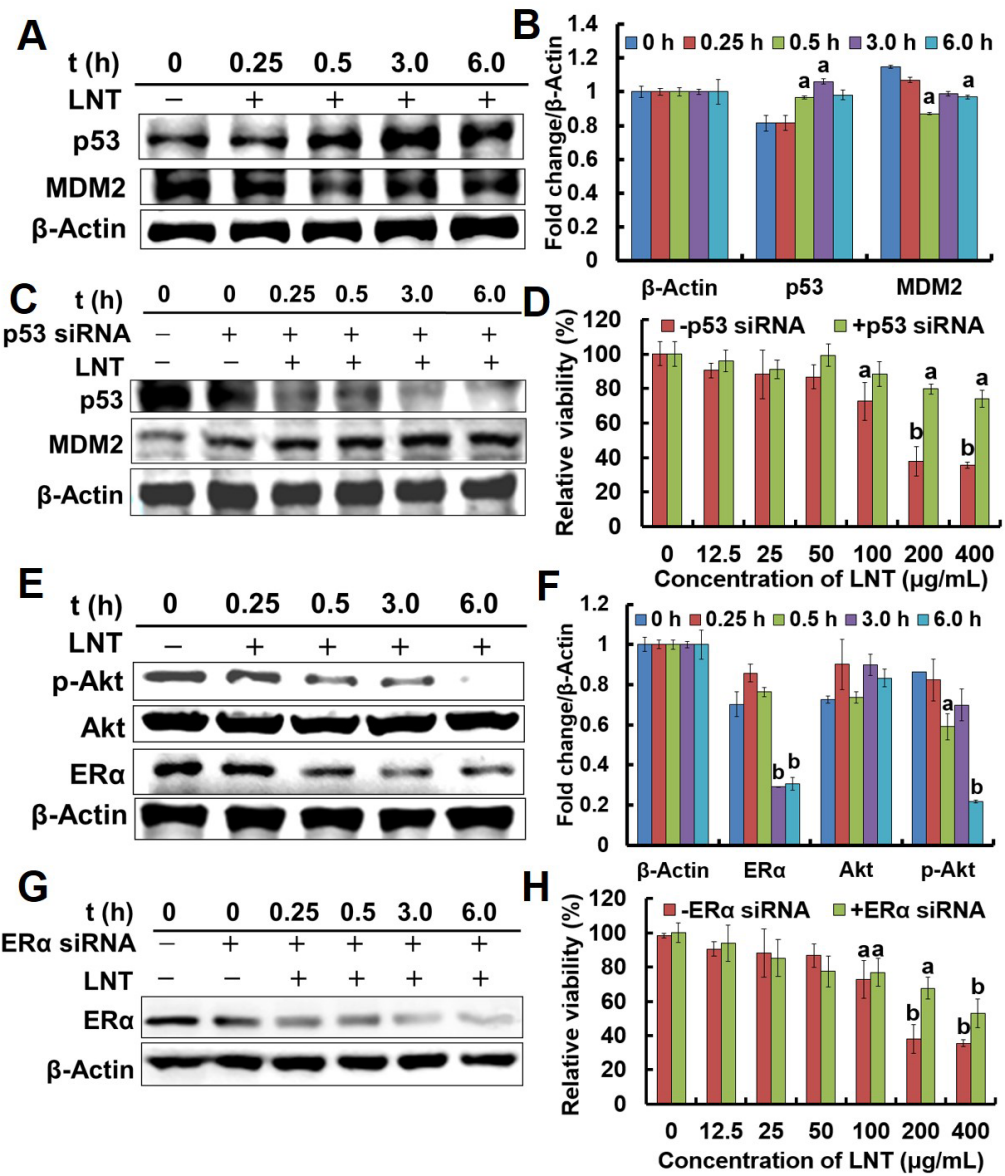
Effects of LNT on tumor cells *in vitro* **(A)** p53 and MDM2 protein expression in MCF-7 cells detected by Western blotting analysis using their specific antibodies with β-Actin as the loading control. MCF-7 cells were treated with 200 μ/mL LNT for indicated time intervals, and the whole cell extracts were prepared, 30 μg of which were resolved on 10% or 12% SDS-PAGE. **(C)** p53 and MDM2 protein expression in p53 siRNA-transfected MCF-7 cells detected by Western blotting analysis using their specific antibodies with β-Actin as the loading control. MCF-7 cells were transfected with p53 siRNA, and treated with LNT (200 μg/mL) for indicated time intervals, followed by extracting the whole cell proteins. The blots are run under the same experimental conditions as in (A). **(D)** Effect of LNT on cell viability in p53 siRNA-transfected MCF-7 cells after incubation for 48 h. Cell viability was determined by MTT assay. **(E)** ERα, p-Akt and Akt protein expression in MCF-7 cells detected by Western blotting analysis using their specific antibodies with β-Actin as the loading control. MCF-7 cells were treated with 200 μ/mL LNT for indicated time intervals, and the whole cell extracts were prepared, followed by Western blotting analysis. **(G)** ERα protein expression in ERα siRNA-transfected MCF-7 cells detected by Western blotting analysis using their specific antibodies with β-Actin as the loading control. MCF-7 cells were transfected with ERα siRNA, and then treated with LNT (200 μg/mL) for indicated time intervals. **(H)** Effect of LNT on cell viability in ERα siRNA-transfected MCF-7 cells after incubation for 48 h. Cell viability was determined by MTT assay. Values expressed are means ± SD of triplicates. **(B, F)** The digital results were determined by quantitative densitometry from three independent experiments. All the protein bands shown are representative of three independent experiments.^a^*p* <0.05 and ^b^*p* <0.001 versus the control.

The gene of p53 is now recognized to be the singly most frequently inactivated tumor suppressor in human cancers [24], which either counters cell proliferation or induces various cell cycle checkpoints, apoptosis or cellular senescence [25]. Mouse double minute 2 (MDM2) is one of cell cycle checkpoints regulated by p53, and it normally acts to block p53 activation through binding to p53 [24]. And inhibition of MDM2 induces p53, leading either to p53-dependent apoptosis or to induction of cell cycle arrest [24]. To further study the mechanism under which LNT induced proliferation inhibition of breast cancer cells, proteins in MCF-7 and T47D ER+ cells were extracted. LNT remarkably enhanced the expression of p53 proteins in MCF-7 cells (Figure 3A and 3B) after a 30 min-treatment in contrast to the control. As expected, the expression of MDM2 proteins was down-regulated after LNT-treatment for 30 min. Similarly, LNT also enhanced p53 protein expression in T47D cells (Supplementary Figure 4).

To clarify the key role of p53 in ER+ breast cancer cells, transfecting p53 siRNA into MCF-7 cells before LNT treatment was performed. Consequently, p53 protein expression was significantly down-regulated after p53 siRNA transfection (Figure 3C), suggesting successful block of the gene of p53. Interestingly, LNT greatly enhanced MDM2 expression in p53 siRNA-transfected MCF-7 cells with increasing treatment time (Figure 3C), explaining the continuous decrease of p53 because MDM2 stimulates p53 to degrade [25]. More importantly, LNT largely deceased the ability to inhibit the cell viability after p53 siRNA transfection at LNT concentrations of 200 and 400 μg/mL (Figure 3D). In other words, the cytotoxicity of LNT to MCF-7 cells decreased due to p53 down-regulation, revealing that LNT inhibited MCF-7 cells proliferation at least partly depending on the p53-dependent pathway.

MCF-7 and T47D cells are ER+ breast cancer cell lines, and endocrine therapy is an important class of target-directed therapy that blocks the growth-promoting effects of estrogen via modulating ER transcription, down-regulating ER expression, and inhibiting estrogen biosynthesis [4]. Herein, LNT noticeably repressed ERα proteins level in MCF-7 (Figure 3E and 3F) and T47D cells (Supplementary Figure 4). ERα expression was then inhibited by transfecting ERα siRNA into MCF-7 cells (coded as ER-MCF-7 cells) before LNT treatment (Figure 3G). Correspondingly, LNT partially decreased the capacity to inhibit the relative viability of MCF-7 cells after ERα siRNA transfection (Figure 3H). In combination with all these data in Figure 3, it is proposed that LNT targeted both p53 and ERα to inhibit MCF-7 cell proliferation *in vitro*.

### LNT suppresses MCF-7 tumor growth through cell proliferation inhibition and cell apoptosis promotion in nude mice

**Figure 4:**
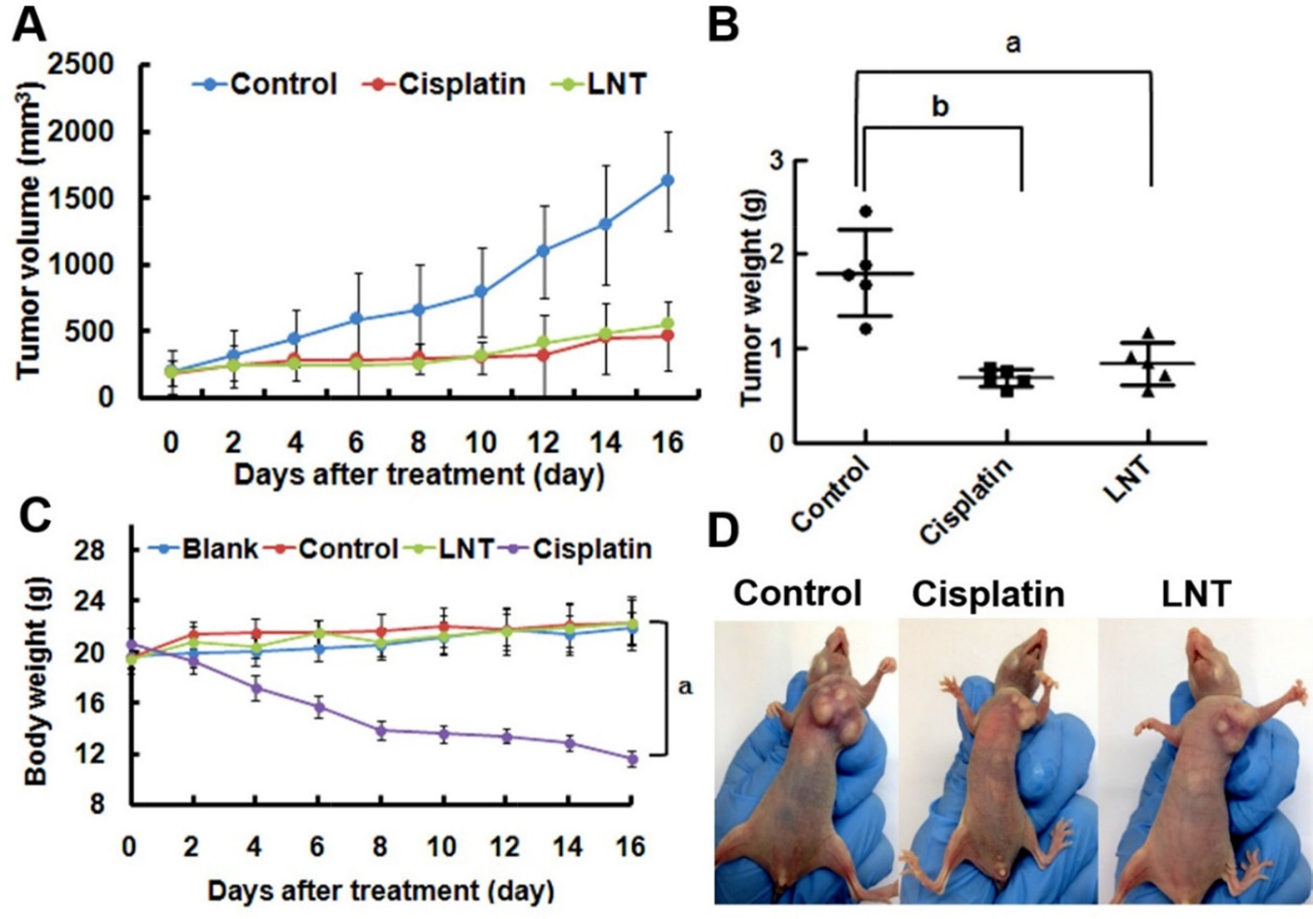
Anti-tumor effects of LNT *in vivo* **(A)** Tumor sizes of the mice as a function of time. **(B)** Tumor weights at the end of the experiment. ^a^*p* <0.05 and ^b^*p* <0.001 versus the control. The data *in vivo* are expressed as means ± SD of 5 mice in each group. **(C)** Body weights of mice after different treatment time. ^a^*p* <0.001 compared to other three groups. **(D)** The photos of MCF-7 tumor-bearing nude mice in different groups.

Based on the *in vitro* results, the effect of LNT on MCF-7 tumor growth in nude mice was investigated. Consequently, LNT led to a similar reduction in tumor volumes (Figure 4A) and tumor weights (Figure 4B) to the positive control of cisplatin; the inhibition ratio was estimated to be ∼53% when compared with the control group of untreated mice. Moreover, there was no significant difference in the body weights in the blank (normal nude mice without tumors), the control (nude mice with tumors) and LNT-treated groups, whereas the body weights of mice in the cisplatin group were significantly reduced (Figure 4C). Figure 4D shows the representative pictures of mice, giving an intuitive effect of LNT on the tumor sizes and body weights of tumor-bearing mice. Clearly, cisplatin led to emaciation of mice due to the well-known serious side effects of chemical drugs, and LNT exhibited invisible cytotoxicity. Additionally, the histological analysis showed that no significant signals of organ damage, inflammatory response, degeneration and necrosis could be detected in LNT group (Supplementary Figure 5). Simultaneously, the clinically used Lentinan injections denoted zs-LNT were used for comparison. Interestingly, LNT exhibited similar to or even higher tumor inhibition than zs-LNT against MCF-7 tumors (Supplementary Figure 6), demonstrating the efficacy of the used LNT in this work.

**Figure 5:**
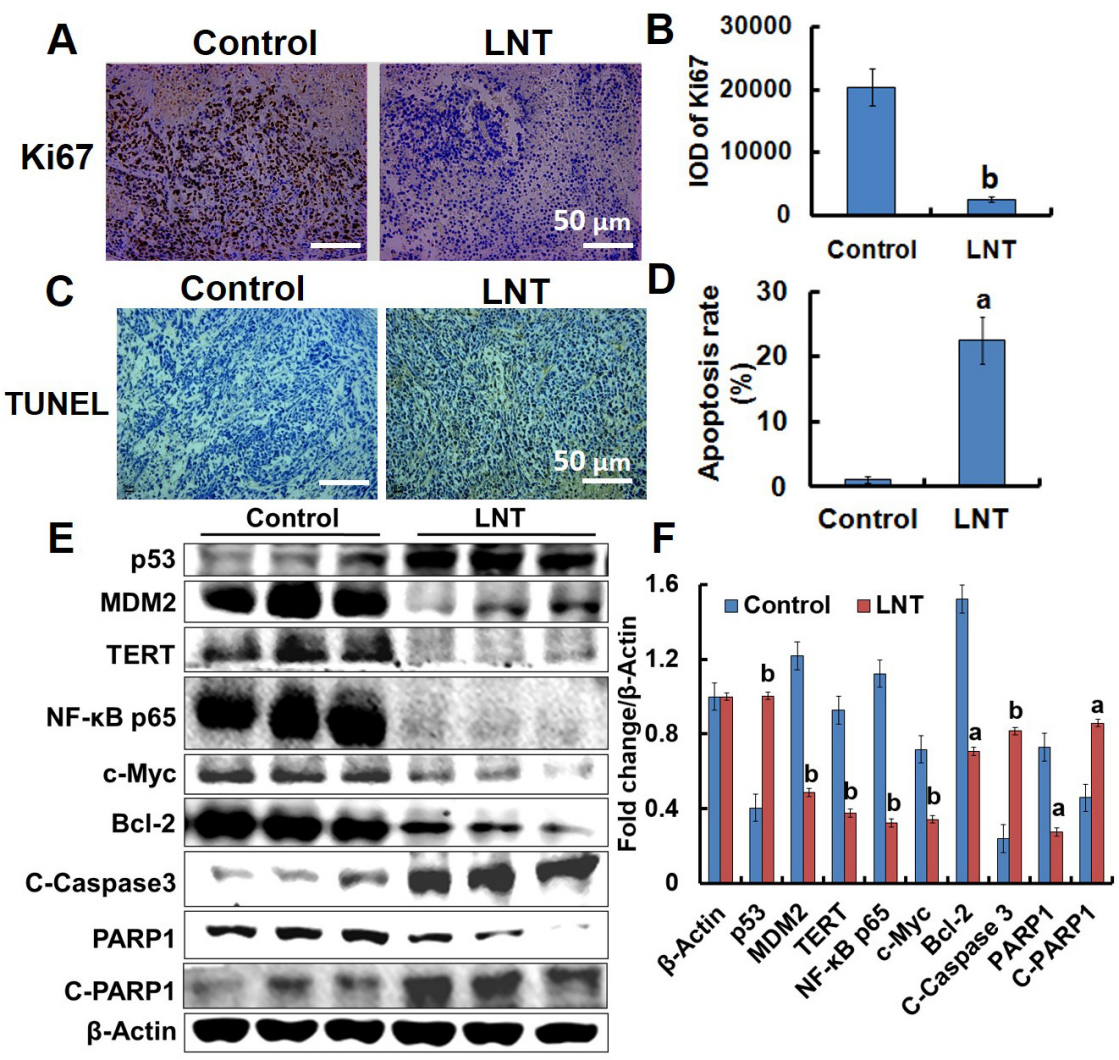
Effects of LNT on tumor cells proliferation and apoptosis in MCF-7 tumor-bearing nude mice **(A, B)** Ki67 (cell proliferation marker) staining in tumor tissues with positive staining for the brown. **(C, D)** TUNEL assay of tumor tissues with positive staining for the brown. Apoptosis rate is estimated as positive cells/total cells. Proportions of positively stained cells were counted in 3 random microscopic fields in each specimen. **(E)** p53, MDM2, TERT, NF-κB p65, c-Myc, Bcl-2, Caspase 3, and PARP1 protein expression in MCF-7 tumors detected by Western blotting analysis using their specific antibodies with β-Actin as the loading control. In each group, the proteins from three mice were used. **(F)** The digital results were determined by quantitative densitometry. All the protein bands shown are representative of three independent experiments. ^a^*p* <0.05 and ^b^*p* <0.001 versus the control. Scale bars, 50 μm.

As shown in the *in vitro* data, LNT inhibited MCF-7 cell proliferation. Therefore, the effect of LNT on the proliferation of cancer cells in mice was investigated. Ki67 is a proliferation marker necessary for cell-cycle progression, replication, and DNA repairing, and TUNEL positive is usually indicative of cell apoptosis [26]. The immunohistochemical images indicated that LNT induced significant reduction in brown color standing for Ki67 staining (Figure 5A and 5B), and an increase of brown color corresponding to the TUNEL-positive cells (Figure 5C and 5D) in MCF-7 tumor tissues. These results incontrovertibly demonstrate that LNT not only suppressed cell proliferation but also induced cell apoptosis in MCF-7 tumor-bearing mice. In combination with the *in vitro* result that LNT primarily inhibited tumor cell proliferation, but did not induce cell apoptosis, it can be suggested that LNT activated immune responses, contributing to cell apoptosis *in vivo*.

### LNT activates the p53- and caspase-dependent pathways in MCF-7 tumors

Since LNT caused tumor growth inhibition through suppressing cell proliferation and promoting apoptosis, proliferation- and apoptosis-related makers and signaling cascades including p53, MDM2, telomerase reverse transcriptase (TERT), nuclear factor-kappa B (NF-κB) p65, c-Myc, Caspase 3, Poly [ADP (ribose)] polymerase 1 (PARP1), B-cell lymphoma-2 (Bcl-2), and phosphorylated extracellular signal-regulated kinase1/2 (p-ERK1/2) were assayed by Western blotting. Clearly, up-regulation of p53 and down-regulation of MDM2 in LNT-treated mice were observed (Figure 5E and 5F), consistent with the *in vitro* data (Figure 3A). Therefore, it is possible that LNT activated p53-dependent pathway to induce either cell cycle arrest or to enhance cell apoptosis via decreasing MDM2 expression. NF-kB transcription factor is widely utilized to regulate genes controlling cell proliferation and cell survival [27], and c-Myc oncogene can promote cell proliferation [24]. The remarkable down-regulation of NF-κB p65 and c-Myc was also seen in LNT-treated MCF-7 tumor tissues (Figure 5E and 5F). Moreover, LNT significantly inhibited phosphorylation of p65 in MCF-7 tumor tissues (Supplementary Figure 7A). The *in vitro* results further demonstrated that LNT not only inhibited NF-κB p65 activation (Supplementary Figure 7B), but also blocked its nuclear translocation (data not shown here). These findings suggested that LNT inhibited MCF-7 tumor growth through cell proliferation suppression via multiple pathways.

Additionally, LNT sharply reduced the levels of anti-apoptotic protein Bcl-2 (Figure 5E and 5F) regulated by p53 [28] and TERT that prevents cancer cells from entering senescence or apoptosis [29], suggesting LNT induced MCF-7 cell apoptosis. RNA-Seq assay showed that LNT up-regulated genes of cytochrome c, somatic (*CYCS*) and tumor protein p53 regulated apoptosis inducing protein 1 (*TP53AIP1*) encoding apoptosis-inducing proteins [30] in MCF-7 tumor tissues (Supplementary Figure 8). The expression of *TP53AIP1* gene is inducible by p53, and it is thought to play an important role in mediating p53-dependent apoptosis [30]. The gene of *CYCS* encoded protein is also involved in initiation of mitochondrion-dependent cell apoptosis. It is thus speculated that LNT-triggered apoptosis in MCF-7 tumor tissues was possibly via p53-dependent and mitochondrial pathways. As well known, caspase-dependent pathway is usually associated with mitochondrion. Interestingly, LNT increased the cleaved Caspase 3 (C-Caspase 3) protein expression and decreased the cleaved PARP1 (C-PARP1) protein level, a hallmark of cell apoptosis occurrence [31, 32], confirming that LNT really promoted tumor cell apoptosis via caspase 3-dependent pathway.

Accumulating evidences indicate that activation of mitogen-activated protein kinases (MAPK) is associated with cell cycle arrest and apoptosis induction. And ERK effectors function as inhibitors of proliferation in MCF-7 cells [33]. As shown in Figure 5A, p-ERK1/2 was really elevated by LNT in MCF-7 tumors. It has been reported that U0126, an inhibitor of mitogen-activated protein kinase kinase (MEK)/ERK signaling significantly inhibits oleanolic acid-induced p53 expression in HepG2 cancer cells [17], suggesting that p53 is regulated by ERK1/2 signaling pathway. We thus speculated that activation of MAPK/ERK/p53 pathway was also responsible for MCF-7 tumor cell proliferation inhibition and apoptosis promotion triggered by LNT in mice.

Taken together, all these findings indisputably suggested that LNT activated p53- and caspase-dependent pathways to inhibit cell proliferation and to promote cell apoptosis, leading to tumor growth inhibition in nude mice.

### LNT suppresses the phosphatidylinositol 3-kinase/protein kinase B/mammalian target of rapamycin (PI3K/Akt/mTOR) pathway and ERα expression in MCF-7 tumors

**Figure 6:**
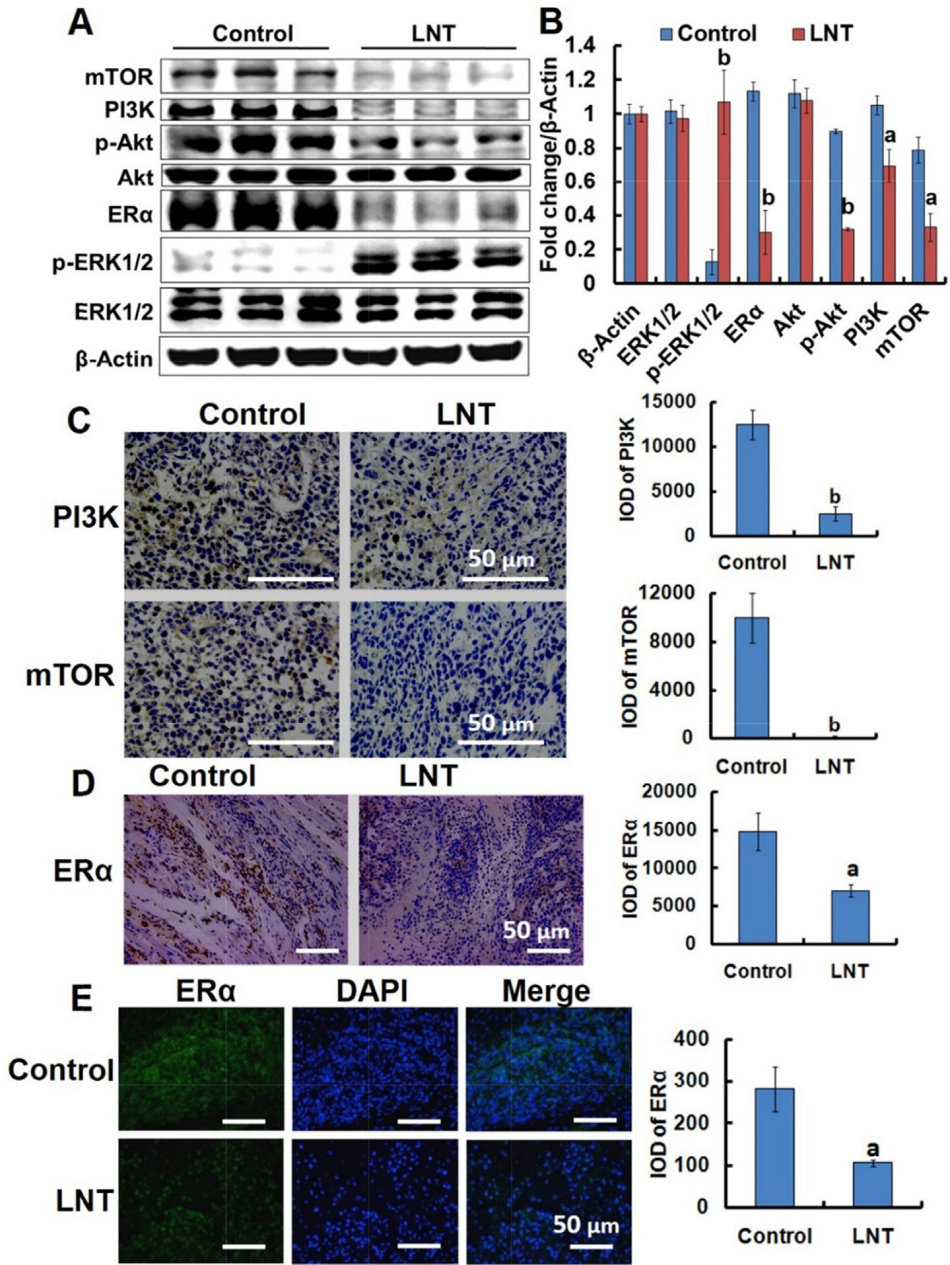
LNT suppresses the PI3K/Akt/mTOR pathway activation and ERα expression in MCF-7 tumor–bearing nude mice **(A)** ERK1/2, p-ERK1/2, ERα, Akt, p-Akt, PI3K and mTOR protein expression in MCF-7 tumors detected by Western blotting analysis using their specific antibodies with β-Actin as the loading control. In each group, the proteins from three mice were used. **(B)** The digital results were determined by quantitative densitometry from (A). ^a^*p* <0.05 and ^b^*p* <0.001 versus the control. **(C)** Immunohistochemical analysis of PI3K and mTOR in MCF-7 tumors. The micrographs were obtained at a magnification of 400 ×. **(D)** Immunohistochemical analysis of ERα in MCF-7 tumors. **(E)** Immunofluorescence assay of ERα in tumor tissues. The micrographs were obtained at a magnification of 200 ×. In panels C and D, proportions of positively stained cells were counted in 3 random microscopic fields in each specimen. ^a^*p* <0.05 and ^b^*p* <0.001 versus the control. Scale bars, 50 μm.

The PI3K/Akt/mTOR signaling pathway is a potentially high relevance to all three major subtypes of breast cancer, driving cell proliferation, growth, and survival [34, 35]. Hyperactivation of this pathway promotes tumor growth and progression frequently found in ER+ breast cancer [4]. We thus explored the effect of LNT on the PI3K/Akt/mTOR pathway in MCF-7 tumor-bearing nude mice. Intriguingly, LNT remarkably decreased the levels of PI3K, phosphorylated-Akt (p-Akt) and mTOR in MCF-7 tumor tissues (Figure 6A and 6B). Immunohistochemical analysis also showed that LNT substantially inhibited PI3K and mTOR expression in LNT-treated tumor tissues compared with the control (Figure 6C). Class I_A_ PI3Ks are the most frequently implicated in breast cancer with mutation and/or amplification of genes encoding the PI3K catalytic subunits p110α (*PIK3CA*), p110β (*PIK3CB*) and p110δ (*PIK3CD*), and the PI3K regulatory subunit p85α (*PIK3R1*), etc. Of these, *PIK3CA* mutations are the most common genetic alterations of this pathway in breast cancer. Genetic or pharmacological inactivation of *PIK3CA* expression results in disappearance of mammary tumors [36]. As shown in Supplementary Figure 8, LNT significantly down-regulated the expression of *PIK3CA* compared with the control, confirming the inactivation of PI3K pathway by LNT. These findings implied that LNT suppressed activation of PI3K/Akt/mTOR pathway in MCF-7 tumors, possibly contributing to inhibition of MCF-7 cell proliferation and survival.

Consistent with the *in vitro* result, LNT significantly decreased ERα expressions in MCF-7 tumors characterized by Western blotting (Figure 5A and 5B) and immunohistochemistry (Figure 5D and 5E), respectively. It is thus concluded that LNT can be used as a down-regulator of ERα, contributing to MCF-7 tumor growth inhibition.

## DISCUSSION

Breast cancer is one of the most common malignancies and has become a serious threat to women’s health worldwide [37]. In this work, we discovered that a naturally occurring β-(1, 3)-D-glucan from *Lentinus edodes* coded as LNT not only selectively inhibited proliferation of MCF-7 and T47D breast cancer cells positively expressing ERα *in vitro*, but also suppressed MCF-7 tumor growth with the inhibition ratio of over 50% via suppressing cell proliferation and promoting cell apoptosis *in vivo*. Furthermore, LNT showed a good safety profile in normal cells and protected the organs from damage compared with the positive control of cisplatin, making it of potential interest in the treatment of ER+ breast cancers.

The strong ability of LNT to efficiently inhibit carcinogenesis in a nontoxic manner thus sparked a great interest in understanding how LNT exerted the anticancer action. As popularly known, tumors are developed from the cells with excessive proliferation out of control, and cell proliferation inhibition is thus one of effective strategies to suppress tumor development and progression. In this respect, LNT is undoubtedly an excellent candidate to inhibit MCF-7 cell proliferation both *in vitro* (Figure 1) and *in vivo* (Figure 5) via cell cycle arrest. As reported, tumor suppressor genes regulate diverse cellular activities such as cell cycle checkpoint responses, detection and repair of damage DNA, etc.[24]. In many human cancers including sarcomas, breast and others, p53 is now recognized to be the most frequently inactivated gene due to blocking p53-dependent transactivation by extra MDM2 genes [25]. MDM2 overexpression contributes to cancer initiation, maintenance or progression [38, 39], and amplification of MDM2 genes or proteins is usually a feature of many tumors that retain wild-type p53. The clinical studies also indicate that p53 reactivation offers an attractive strategy for cancer therapy [40]. The inactivated p53 is characterized by the decreased level due to degradation. MDM2 is such a protein binding to p53, leading to p53 degradation. So inhibition of MDM2 leads to either p53-dependent apoptosis or to cell cycle arrest [25]. In our findings, higher MDM2 expression and lower p53 protein level were observed in MCF-7 cells (Figure 3A and 3B) and tumors (Figure 5E and 5F) without LNT treatment. However, LNT treatment significantly enhanced p53 protein expression and down-regulated MDM2 protein level. Furthermore, the ability of LNT to suppress the proliferation of p53 siRNA-transfected MCF-7 cells was clearly reduced in contrast to the non-transfected cells (Figure 3D). It is thus concluded that LNT targeted p53 to trigger the inhibitory effect on MCF-7 cell proliferation *in vitro*. Moreover, the tumor protein p53 regulated apoptosis inducing protein 1 (*TP53AIP1*) gene, which has been discovered to be directly activated by p53 [30], was up-regulated (Supplementary Figure 8). And the anti-apoptotic protein of Bcl-2 was overexpressed and significantly down-regulated by LNT in MCF-7 tumors (Figure 5E and 5F). It is speculated that LNT inhibited MCF-7 cell proliferation and enhanced cell apoptosis possibly through activating the tumor suppressor p53 via down-regulating the oncogene MDM2.

It has been reported that the activated PI3K/Akt pathway activates MDM2 to terminate the p53 response, leading to tumor growth and survival [4]. Moreover, the PI3K/Akt/mTOR pathway is a key intracellular signaling system that drives cellular growth, survival, differentiation and metabolism [41]. It is the most frequently activated signaling pathway in breast cancers [42], contributing to promoting tumor growth and progression [43]. As reported, activation of PI3K/Akt pathway induces cell proliferation in MCF-7 cells [33]. Therefore, suppression of PI3K/Akt pathway activation was thus expected to involve in MCF-7 tumor growth/cell proliferation inhibition. As well known, PI3K activates Akt via phosphorylation, and the activated Akt stimulates the mTORC1 complex, a key regulator of cellular growth and protein synthesis that includes mTOR and a regulator binding partner [4]. That is, Akt activation will enhance mTOR expression. In this work, after treatment with LNT, the levels of PI3K, p-Akt and mTOR proteins levels were effectively suppressed in MCF-7 tumors (Figure 6A-6C). Interestingly, LNT also reduced the p-Akt level in MCF-7 cells (Figure 3E and 3F). These findings thus suggested that LNT inhibited PI3K/Akt/mTOR and PI3K/Akt/MDM2/p53 pathways, leading to MCF-7 tumor growth/cell proliferation inhibition in nude mice.

NF-kB signaling pathway is a complex network linking extracellular stimuli to cell survival and proliferation, which is precisely regulated in normal tissues, but is prone to innumerable points of dysregulation in cancer and plays a fundamental role in tumorigenesis [27]. As such, aberrant activation of NF-kB has been implicated in the propagation of many cancers [44]. As reported, NF-kB p65 is found to overexpress in tumor tissues compared with the p65 level in paracancerous and normal tissues [45]; blocking NF-kB can cause tumor cells to stop proliferating, to die, or to become more sensitive to the action of antitumor agents [27]. In our findings, LNT remarkably decreased not only the level of p65 protein (Figure 5E), but also inhibited activation of p65 (phosphorylation of p65, p-p65) in MCF-7 solid tumors and cells (Supplementary Figure 7), which also possibly contributing to the cell proliferation inhibition and apoptosis enhancement in mice. It has been reported that NF-κB is regulated by the upstream signaling of Akt [24] and Akt/mTOR [46] in cancers. However, the exact molecular mechanism leading to NF-kB activation in many cancers is not fully known [27].

Increasing evidences show that activation of MAPK is closely associated with cell cycle arrest involved in cell proliferation as well as cell apoptosis [47, 48]. Of three MAPK pathways, ERK1/2 is most relevant to breast cancer [48]; prolonged activation of MAPK/ERK cascade effectively inhibits proliferation in MCF-7 cells [33] due to its stimulation of the synthesis of p21, an inhibitor of cell-division cycle [49, 50]. Herein, weak p-ERK signals were seen in MCF-7 tumors, but LNT treatment remarkably enhanced the level of p-ERK1/2 protein in MCF-7 tumors (Figure 6A and 6B), contributing to significant proliferation inhibition observed in Ki67 staining of MCF-7 tumor tissues (Figure 5A and 5B). More interestingly, there is a significant level of cross-talk between kinases of PI3K-Akt and ERK pathways in both physiological and pathological conditions; inhibition of one cascade activates the other, and vice versa [34]. As reported, inhibition of PI3K and Akt promoted ERK activation in MCF-7 cells [51]. Our recent work reported that LNT enhances p21 expression in S-180 cells and tumors mediated by p53 [14]. Wang et al attributed the inhibitory effect of oleanolic acid on hepatocellular carcinoma to ERK-p53-mediated cell cycle arrest and mitochondrial-dependent apoptosis [17]. In combination with these data and analysis, we thus suggested that LNT-induced ERK-p53 pathway activation, leading to cell proliferation inhibition. In contrast, the other two MAPK pathways of c-Jun N-terminal protein kinase (JNK) and p38 are stress-activated pathways with pleiotropic roles of anti-proliferative and proapoptotic effects as well as cancer promoters depending on cell type, nature of the death stimulus, duration of its activation and the activity of other signaling pathways [52–54]. As shown in Supplementary Figure 7A, LNT significantly inhibited JNK activation, but hardly affected p38 activation. It is possible that JNK and p38 pathways are not the major ones involved in MCF-7 tumor inhibition.

Caspase family often functions as vital components of the apoptotic machinery and acts to destroy specific target proteins which are critical to cellular longevity [55]. The cleaved Caspase 3 was observed to be remarkably up-regulated (Figure 5E and 5F), which is proteolytically generated during apoptosis from the inactive caspase precursor [56]. In particular, PARP1 expression was down-regulated, which is cleaved by Caspase 3 as the actual marker of cell apoptosis *in vivo* [57]. Therefore, up-regulation of the cleaved Caspase 3 and down-regulation of the cleaved PARP1 demonstrate that the actual occurrence of apoptosis in MCF-7 tumors initiated by LNT. Taken together, LNT triggered p53- and caspase-dependent apoptosis in MCF-7 breast tumors, leading to tumor growth inhibition.

As reported, down-regulation of ERα is one of the mechanisms of target-directed endocrine therapy [4]. Herein, ERα expression was sharply down-regulated *in vitro* (Figure 3E and 3F). When ERα siRNA was transfected into MCF-7 cells *in vitro* (Figure 3G), the inhibitory effect of LNT on the viability of MCF-7 cells was correspondingly reduced (Figure 3H), suggesting LNT targeted ERα to inhibit MCF-7 cell proliferation. Similar to the data *in vitro*, the results from Western blotting showed that LNT clearly down-regulated ERα *in vivo* (Figure 6A and 6D), suggesting that LNT is a good intervention in ER+ breast cancer endocrine therapy. However, the exact correlation of LNT to ERα remains unclear. More interestingly, β-glucan receptors of dectin-1 and CR3 expressed on the MCF-7 cell surface, and LNT bound to the cell surface. However, blocking of dectin-1 and CR3 did not affect the cell proliferation inhibition triggered by LNT. These findings suggested that LNT possibly initiated some other unknown receptor- but not dectin-1 or CR3-dependent pathways to repress tumor cell proliferation.

It is worth noting that LNT was intraperitoneally injected into mice, and LNT would not directly interact with tumor cells. But LNT similarly induced cell proliferation inhibition and apoptosis promotion in mice, implying that it is the immune responses stimulated by LNT that caused tumor growth *in vivo*. These results were similar to our previous work that LNT may induce tumor cell apoptosis through activating immune responses *in vivo* [14].

**Figure 7:**
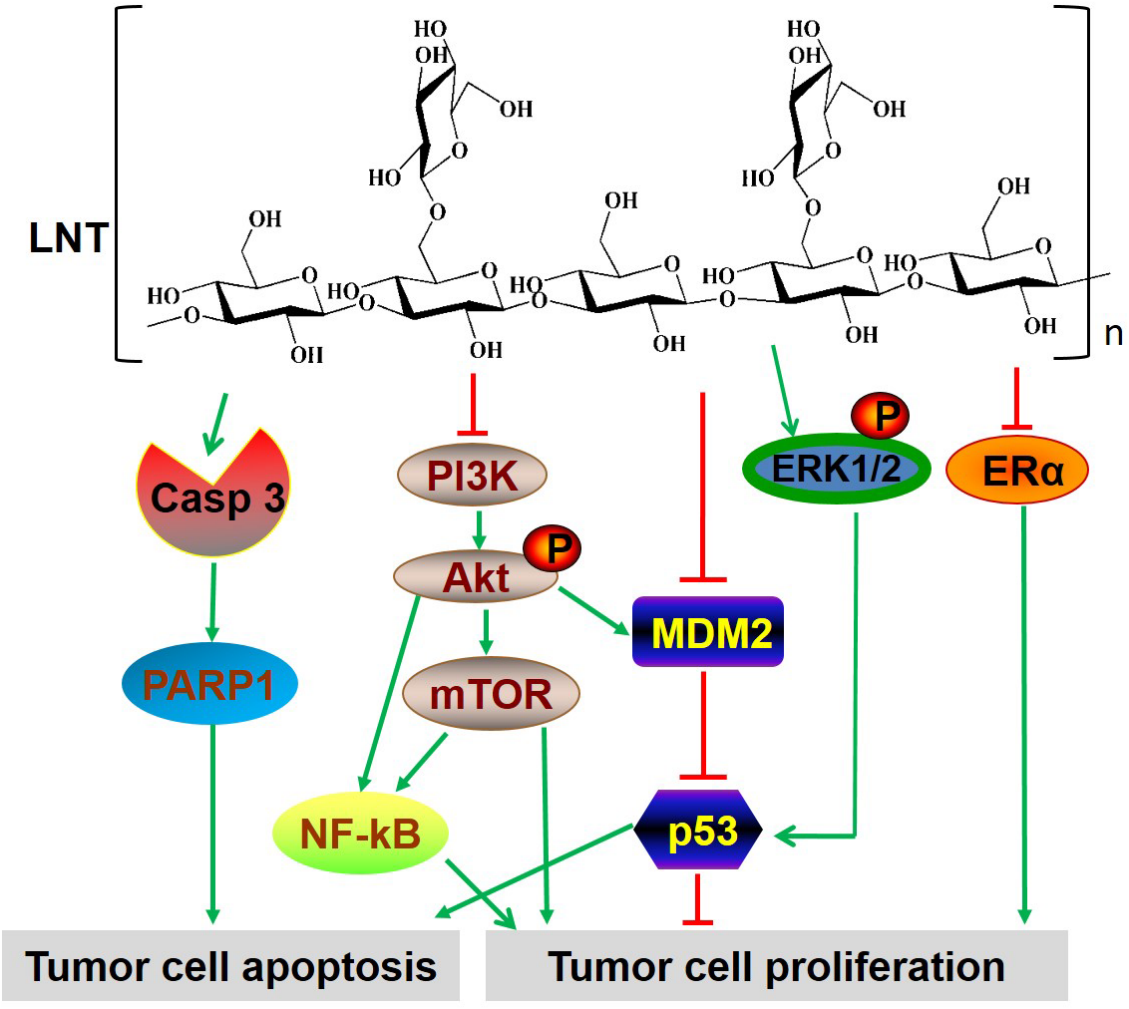
The multiple signaling pathways possibly involved in LNT-treated ER+ MCF-7 breast tumor tissues

In conclusion, all these findings *in vivo* and *in vitro* assays evidently demonstrated that LNT showed significant anti-tumor effects without toxicity. The possibly involved mechanisms by which LNT inhibited MCF-7 breast tumor growth were proposed as follows. (1) LNT interacts with MCF-7 cells and induces cell cycle arrest at G2/M phase via MDM2/p53- and ERα-dependent pathways, leading to cell proliferation inhibition *in vitro*; (2) LNT suppresses activation of PI3K/Akt/MDM2/p53 and PI3K/Akt/mTOR pathways, as well as activates the ERK-dependent pathway, responsible for MCF-7 cell proliferation inhibition and apoptosis promotion *in vivo* (Figure 7); (3) LNT activates caspase 3-dependent signaling pathway to induce tumor cell apoptosis *in vivo* (Figure 7). Hence, LNT can be considered as a potential anticancer drug for the effective treatment of ER+ breast cancers through multiple signaling pathways. This work provides a novel strategy to treat ER+ breast cancers by using a naturally occurring β-glucan from mushrooms.

## MATERIALS AND METHODS

### Beta-glucan samples

The beta-glucan (LNT) was isolated from the dried fruiting bodies of *Lentinus edodes*, following the procedures described in our previously reported work [14]. The total sugar content of LNT was determined to be 98.7% by using the classic colorimetric method for determination of sugars and related substances [58]. The protein content was analyzed to be 0.52% by the method of Bradford using bovine serum albumin (BSA, Sigma) as the standard. The endotoxin was determined by the chromogenic limulus amebocyte lysate assay. It was found there was no detectable level of endotoxin in LNT samples. All these data showed that LNT used in this work was of high purity. The viscosity-average molecular weight of LNT in water was determined to be 8.9×10^5^ by a viscometer. The molecular weight range of LNT was determined by laser light scattering combined with size-exclusion chromatography (Supplementary Figure 9) to be 50×10^4^-200×10^4^. LNT was dissolved in the cell culture medium or PBS for *in vitro* assays, and in the saline for *in vivo* assays, which was sterilized at 121°C for 20 min and kept at 4°C before use.

### Cell lines and animals

The human breast cancer cell lines of ER+ MCF-7 and T47D, triple-negative cell lines of MDA-MB-231 and MDA-MB-468 lacking in receptors of estrogen, progesterone and human epidermal growth factor (ER, PR, HER2), and the human normal cell lines including breast cell (HBL-100), hepatocyte cell (LO2) and embryonic kidney 293T cell were purchased from China Center for Typical Culture Collection (CCTCC, Wuhan, China). LO2 cells were cultured in the altered RPMI 1640 medium (HyClone) supplemented with penicillin (100 U/mL), streptomycin (100 μg/mL), and 10% heat-inactivated fetal bovine serum (FBS, Gibco). All other cells were routinely grown in Dulbecco’s modified Eagle’s medium (DMEM, high glucose, HyClone) supplemented with 10% heat-inactivated FBS, 100 U/mL penicillin and 100 μg/mL streptomycin. All cell lines were incubated at 37°C under a humidified atmosphere of 95% air and 5% CO_2_.

Female athymic BALB/c nude mice (6–8 weeks old), obtained from Hunan Slack Scene of Laboratory Animal Co., LTD (Hunan, China) were used. All the animals were kept at room temperature at 25 ± 2°C, 12 h dark–light cycle and a relative humidity of 60–70%. All the mice were fed with normal mice chow and water ad libitum under specific pathogen free (SPF) conditions. All animal experiments were performed in accordance with the institutional guidelines for Laboratory Animal Care approved by the Institutional Ethical Committee of Wuhan University.

### Cell viability/proliferation assay *in vitro*

Cell viability was determined by using a standard 3-(4, 5-dimethyl-2-thiazolyl)-2, 5- diphenyl-2-H-tetrazolium bromide (MTT) assay [59]. Briefly, exponentially growing cells in 96-well plates were treated with LNT at different concentrations (0-400 μg/mL) in the complete medium. 20 μL of MTT (5 mg/mL, Sigma-Aldrich, USA) was added to each well after treatment for designed times as indicated in the figure. After the plates were incubated at 37°C for another 4 h, the supernatant was aspirated, and 200 μL of dry dimethyl sulfoxide (DMSO, Sinopharm, China) was added. Absorbance was measured at 570 nm by a 96-well microplate reader (BMG LABTECH, FLUOstar OPTIMA, Germany). The percentage of surviving cells was calculated as follows: relative viability (%) = (mean absorbency in test wells) / (mean absorbency in control wells) × 100.

For the cell proliferation assay, exponentially growing cells were seeded in 96-well plates at a density of 6×10^3^ cells/well and incubated with LNT at various concentrations (0-400 μg/mL) for a desired time as indicated in the figure. Cell proliferation was measured by counting the total number of living cells with the Hemocytometer. Trypan blue dye-exclusion assay was performed to observe cell death. All experiments were performed in triplicate.

### Cellular uptake of LNT by MCF-7 cells

LNT was first labeled with the fluorescein isothiocyanate isomer I (FITC, Sigma, US) through covalently reacting with hydroxyl groups according to the reported procedure [60]. Briefly, LNT (200 mg), FITC (30 mg), pyridine (100 μL, Sinopharm, China) and dibutyltin dilaurate (20 μL, Sinopharm, China) were first dissolved in DMSO (20 mL, Sigma, US). The reaction mixture was heated for 4 h at 100°C and precipitated with 4 volumes of ethanol followed by centrifugation (6000 rpm, 10 min). The precipitation was repeated four times in total to remove the unbound FITC. The FITC-labeled LNT, denoted FITC-LNT, was finally obtained after drying at 60°C, and was kept in dark before use.

The qualitative interaction of FITC-LNT was monitored with a laser-scanning confocal microscope (Nikon C1-si TE 2000, Japan). MCF-7 cells were seeded into the confocal dish at a density of 2×10^5^ cells/dish for 24 h, followed by incubation with FITC-LNT (200 μg/mL) for 40 min at 4°C. At the end of incubation, the medium was removed, and the cells were rinsed three times with PBS. The nuclei were then stained with Hoechst 33342 (10 μg/mL) for 20 min at room temperature in dark. Then, the cells were washed two times with PBS followed by fixing with 1 mL of 4% paraformaldehyde for 10 min. Finally, the stained cells were subjected to confocal microscopy observation after washing with PBS.

For quantitative estimation of LNT attached to cells or its cellular uptake, MCF-7 cells were separately seeded into a six-well plate at a density of 3.0×10^5^ cells/well and cultured with 2 mL of DMEM containing 10% heat-inactivated FBS for 24 h, followed by incubation with FITC-LNT (200 μg/mL) for the desired time at 37°C for 2 h or at 4°C for 40 min. The cells were washed three times with PBS, and treated with RIPA buffer (50 mM Tris-HCl, 150 mM NaCl, 1% NP-40, 0.5% sodium deoxycholate, 0.1% SDS, 1 mM PMSF, and 1 mM EDTA, pH 7.4) on ice for 20 min. After a 5-min centrifugation at 14000 rpm, the supernatant was saved as cell lysates. Finally, the fluorescence intensities of cell lysates were measured with excitation at 488 nm and emission at 525 nm using a microplate reader (TECAN, SPARK 10M, Austria).

### Cell cycle analysis *in vitro*

Cell cycle was studied by measuring the DNA content of nuclei labeled with propidium iodide (PI). MCF-7 cells were treated with varying concentrations of LNT. After treatment for 24 h, cells were harvested by centrifugation, washed with ice-cold PBS and fixed in 70% cold ethanol at 4°C for 12 h. Thereafter, cells were washed twice and stained with RNase (10 μg/mL) and PI (50 μg/mL) for 30 min at 37°C in dark. Cell cycle distribution was performed using a flow cytometer (BD FACSVerseTM, USA) and the percentages of cells at G1, S and G2/M phases were calculated (FlowJo 6.0 software).

### Anti-breast cancer assay *in vivo*

Exponentially growing MCF-7 cells suspended in PBS were injected subcutaneously into the left flanks of nude mice (5×10^6^ cells in 100 μL). When the size of established tumors reached about 200 mm^3^ (around two weeks after tumor cells were inoculated), 15 mice were randomized into three groups (n = 5) followed by receiving a daily intraperitoneal injection of 0.9% NaCl (negative group, 15 days), 2.5 mg/kg cis-platinum (positive group, every three days) and 1 mg/kg LNT (LNT group) in a 200 μL volume, respectively. The remaining five mice was set as the blank control (normal mice without MCF-7 cancer cells transplantation). Tumor sizes of mice were measured every two days before sacrifice. The tumor volume was calculated by the formula of tumor volume (mm^3^) = length × width^2^/2 [61]. The mice were finally killed and the local tumors were removed carefully. Each tumor was split into two halves: one half was fixed in 4% buffered, freshly prepared paraformaldehyde, embedded in paraffin, and made into paraffin sections (5 mm thickness) for histological and immunohistochemical observation; the other one was stored at -80°C before use.

### Western blotting

The total proteins of MCF-7 or T47D cells were extracted according to the following procedures. Cells were plated in a 60-mm culture dish at a density of 3×10^6^ cells/dish in the culture medium with 10% FBS for 24 h, and then treated with or without LNT under different conditions; at the end of treatment, the cells were harvested and treated with RIPA buffer containing 1 mM PMSF on ice for 20 min. After a 5-min centrifugation at 14000 rpm, the supernatant was saved as the total protein extract. The MCF-7 tumor tissues were cut into small pieces and lysed by the same RIPA buffer as described above on ice for 20 min; after a 5-min centrifugation at 14000 rpm, the supernatant was harvested as the tumor tissue protein extract. Both cell and tumor tissue proteins were preserved at -80°C for Western blotting assay. The protein concentrations were quantified using a BCA protein assay Kit (Beyotime Institute of Biotechnology) with bovine serum albumin (BSA) as the reference.

The cell or tumor tissue lysates were mixed with 4 × SDS sample buffer and denatured in boiling water for 5 min. Aliquots of 20∼60 μg of denatured total proteins were separated by SDS-PAGE on a 12% or 10% polyacrylamide gel and then electrically transferred onto a PVDF membrane (0.45 μm, Millipore). After blocking with 5% (w/v) BSA in TBS (10 mM Tris-HCl (pH 8.0) and 150 mM NaCl) containing 0.1% Tween 20 at room temperature for 1 h, the membranes were then incubated with the corresponding specific primary antibodies including Bcl-2, p53, MDM2 (SMP14), Caspase 3, PARP1, TERT (H-231), c-Myc (N-262), NF-κB p65 (C-20), Akt (H-136), p-Akt (Ser474), ERK1/2 (H-72), p-ERK1/2 (Thr177/Thr160), ERα (HC-20), PI3K p110α (H-201), and mTOR (H-266) overnight at 4°C. To ensure that equal amounts of proteins had been loaded into each lane of the SDS gel, the antibody against β-Actin (I-19) was used as the loading control. Antibodies were obtained from the following sources: Bcl-2 antibody, Abcam; Caspase3 and p53 antibodies, Cell Signaling Technology; PARP1, ABclone; and all others, Santa Cruz Biotechnology. The reactive bands were visualized with a horseradish peroxidase (HRP)-conjugated secondary antibody (Biosharp) for 50 min via enhanced chemiluminescence (ECL) Western blotting detection reagent on a ChemiDoc-It™ imaging system (UVP, America) according to the manufacturer’s instructions. The images were quantified by densitometric analysis using the Quantity One software. Relative intensities were normalized through dividing the average gray value of the target protein by the average gray value of the corresponding β-Actin.

### Knockdown of p53 and ERα with siRNA *in vitro* MCF-7 cells

MCF-7 cells were seeded into a 96-well plate or a 60 mm culture dish in DMEM with 10% FBS. After a 24 h pre-incubation, the cells were transfected with p53 siRNA and ERα siRNA (Santa Cruz Biotechnology) using Lipofectamine 2000 (Invitrogen) according to the manufacturer’s protocols. After a 12-h transfection, the cells were rinsed with PBS and incubated in DMEM containing 10% FBS for another 48 h. In 96-well plates, the cells were then incubated with LNT dissolved in the complete cell culture medium or PBS at different final concentrations of 0-400 μg/mL. After incubation for 48 h, cell viability was determined by using MTT assay. In the case of dishes, the cells were then stimulated with LNT (200 μg/mL) for desired time as indicated in the figure. At the end of treatment, the cells were rinsed with PBS and harvested for the total protein extraction according to the same procedures as described above. The cell lysates were collected for Western blotting analysis.

### Histological and immunohistochemical assays

For hematoxylin and eosin (HE) staining, all specimens were fixed in 10% formalin for 24 h and then embedded in paraffin followed by sectioning into 4-mm slices. The histological sections were stained with HE and observed under a light microscope. Immunohistochemistry was performed according to the manufacturer’s instructions (LSAB kit; Dako, Carpinteria, CA, USA). In brief, tumor sections were cut and deparaffinized in xylene, dehydrated in the graded alcohol and finally hydrated in water; antigen retrieval was performed by boiling the slide in 10 mM sodium citrate (pH 6.0) for 30 min; slides were then incubated overnight with primary antibodies including anti-Ki67, anti-ERα, anti-PI3K and anti-mTOR (each at 1:200 dilution). Apoptotic cells were detected using a terminal deoxynucleotidyl transferase-mediated dUTP nick end labeling (TUNEL) apoptosis detection kit (Millipore, USA) according to the manufacturer’s instructions. These slides were subsequently washed several times in Tris-buffered saline with 0.1% Tween-20 and incubated with biotinylated linker for 30 min, followed by incubation with streptavidin conjugate provided in LSAB kit (Dako) according to the manufacturer’s instruction. Immunoreactive species were detected using 3, 3-diaminobenzidine tetrahydrochloride as a substrate; sections were counterstained with Gill’s haematoxylin and mounted under glass coverslips. Images were taken using a microscope (NIKON ECLIPSE TI-SR, Japan; Leica Dmi8, Germany). For quantitation, images were acquired from three different fields at 200 × magnification, and cells were counted using Image pro-plus 6.0 software.

### Immunofluorescence staining

Cryosections of tumors were stained with anti-ERα (Santa Cruz) antibody, followed by a biotinylated secondary antibody (Santa Cruz) and streptavidin-FITC (fluorescein isothiocyanate) with 4, 6-diamidino-2-phenylindole (DAPI; Invitrogen) counterstaining to detect tumor vasculature. The fluorescence images were taken by a microscope (NIKON ECLIPSE TI-SR, Japan), and were processed by using Image pro-plus 6.0 software.

### Statistical analysis

All experiments were performed independently and repeated at least two times. Data from the experiments were presented with means ± standard deviation (SD). Statistical comparisons of data sets were evaluated by the student *t*-test.

## SUPPLEMENTARY MATERIALS FIGURES



## Abbreviations

(ER+): Estrogen receptor positive
(ER-): Estrogen receptor negative
(ERα): Estrogen receptor α
(ER): Estrogen receptor
(PR): Progesterone receptor
(HER2): Human epidermal growth factor receptor 2
(ERK1/2): Extracellular signal-regulated kinase1/2
(p-ERK1/2): Phosphorylated-ERK1/2
(MDM2): Mouse double minute 2
(PI3K): Phosphatidyl inositol 3-kinase
(Akt): Protein kinase B
(p-Akt): Phosphorylated-Akt
(mTOR): Mammalian target of rapamycin
(TUNEL): Terminal-deoxynucleotidyl transferase mediated nick end labeling
(NF-κB): Nuclear factor-kappa B
(TERT): Telomerase reverse transcriptase
(PARP1): Poly-ADP-ribose polymerase 1
(Bcl-2): B-cell lymphoma-2
(CYCS): Cytochrome c, somatic
(TP53AIP1): Tumor protein p53 regulated apoptosis inducing protein 1
(PAMP): Pathogen associated molecular pattern
(MTT): 3-(4, 5-dimethyl-2-thiazolyl)-2, 5- diphenyl-2-H-tetrazolium bromide
(FITC): Fluorescein isothiocyanate isomer I
(PI): Propidium iodide
(SDS): sodium dodecyl sulfate
(BSA): bovine serum albumin
(BSA): fetal bovine serum x
(DMEM): Dulbecco’s modified Eagle’s medium
(JNK): c-Jun N-terminal protein kinase
