# Supplementary material for: The β-glucan from Lentinus edodes suppresses cell proliferation and promotes apoptosis in estrogen receptor positive breast cancers
Source: Oncotarget. 2017 Sep 30;8(49):86693–709. doi: 10.18632/oncotarget.21411 (PMC5689718; doi:10.18632/oncotarget.21411)
Supplement: Supplementary file 1 [file oncotarget-08-86693-s001.pdf]

The β-glucan from *Lentinus edodes* suppresses cell proliferation and promotes apoptosis in estrogen receptor positive breast cancers

SUPPLEMENTARY MATERIALS

RNA processing, microarray hybridization and data analysis

Raw and normalised gene expression files are available from BGI Tech (China). Total RNA was extracted from tumor tissues using RNAeasy mini kit (Qiagen). There were two methods to treat total RNA. Oligo (dT) magnetic beads were used to select mRNA with polyA tail, or hybridized the rRNA with DNA probe and digested the DNA/RNA hybrid strand, followed by DNase I reaction to remove DNA probe. Then the target RNA after purification was obtained. Fragmented the target RNA and reversed transcription to double-strand cDNA (dscDNA) by N6 random primer. Ended repair the dscDNA with phosphate at 5' end and stickinessed 'A' at 3' end, then ligate and adaptor with stickiness 'T' at 3' end to the dscDNA. Two specific primers were used to amplify the ligation product. The PCR product was denatured by heat and the single strand DNA was cyclized by splint oligo and DNA ligase. Arrays were scanned using an Illumina iScan (Illumina). After getting raw data, each bioinformitics analysis were done as the client appoints on contract. We used Bowtie2 [1] to map clean reads to reference gene and use HISAT [2] to reference genome. Gene Quantification was analyzed by RSEM [3]. Differential gene expression analysis was performed using NOISeq method [4] between control and treated groups. Functional enrichment analysis of differentially expressed genes was performed using KEGG [5]. Heatmaps of differentially expressed genes belonging to clusters enriched were generated using log2 fold change expression values calculated between control and treated conditions. Two samples of each group were used to experiment.

Confocal microscopy

MCF-7 cells were seeded into the confocal dish at a density of 2×10^5 cells/dish for 24 h, followed by incubation at 4°C for 40 min with PE-conjugated anti-dectin-1 antibody (RH1; Bio-legend; San Diego, USA), and PE-conjugated anti-CD11b (M1/70; Biolegend; San Diego, USA). At the end of incubation, the medium was removed, and the cells were rinsed three times with PBS. The nuclei were then stained with Hoechst 33342 (10 μg/mL) for 20 min at room temperature in the dark. Then, the cells were washed two times with PBS followed by fixing with 1 mL of 4% paraformaldehyde for 10 min. Finally, the stained cells were subjected to confocal microscopy observation after washing with PBS using laser excitation at 405, 488 and 543 nm.

Western blotting

The cell or tumor tissue lysates were mixed with 4 × SDS sample buffer and denatured in boiling water for 5 min. Aliquots of 20~60 μg of denatured total proteins were separated by SDS-PAGE on a 12% or 10% polyacrylamide gel and then electrically transferred onto a PVDF membrane (0.45 μm, Millipore). After blocking with 5% (w/v) BSA in TBS (10 mM Tris-HCl (pH 8.0) and 150 mM NaCl) containing 0.1% Tween 20 at room temperature for 1 h, the membranes were then incubated with the corresponding specific primary antibodies including phosphorylated NF-κB p65 (p-p65), p-JNK and p-p38 overnight at 4°C. The antibodies against β-Actin (I-19) and glyceraldehyde-3-phosphate dehydrogenase (GAPDH) were used as the loading control. The reactive bands were visualized with a horseradish peroxidase (HRP)-conjugated secondary antibody (Biosharp) for 50 min via enhanced chemiluminescence (ECL) Western blotting detection reagent on a ChemiDoc-It™ imaging system (UVP, America) according to the manufacturer's instructions.

Anti-breast cancer assay *in vivo*

Exponentially growing MCF-7 cells suspended in PBS were injected subcutaneously into the left flanks of nude mice (5×10^6 cells in 100 μL). When the size of established tumors reached about 200 mm^3 (around two weeks after tumor cells were inoculated), 15 mice were randomized into three groups (n = 5) followed by receiving a daily intraperitoneal injection of 0.9% NaCl (negative group, 15 days), 1 mg/kg LNT (LNT group) and zs-LNT (clinically used LNT injections, zs-LNT group) in a 200 μL volume, respectively. Tumor sizes of mice were measured every two days before sacrifice. The tumor

volume was calculated by the formula of tumor volume (mm^3) = length × width^2/2. The mice were finally killed and the local tumors were removed carefully for weighing.

REFERENCES

1. Langmead B, Trapnell C, Pop M and Salzberg SL. Ultrafast and memory-efficient alignment of short DNA sequences to the human genome. Genome Biol 2009; 10: 25-34.
2. Kim D, Langmead B and Salzberg SL. HISAT: a fast spliced alignerwith lowmemory requirements. Nat Methods 2015; 12: 357-360.
3. Li B and Dewey CN. RSEM: accurate transcript quantification from RNA-Seq data with orwithout a reference genome. BMC bioinformatics 2011; 12: 323.
4. Tarazona S, Garcı´a-Alcalde F, Dopazo J, Ferrer A and Conesa A. Differential expression in RNA-seq: A matterof depth. Genome Res 2011; 21:2213-2223.
5. Kanehisa M. KEGG forlinking genomes to life and the environment. Nucleic Acids Res 2008; 36: D480-484.

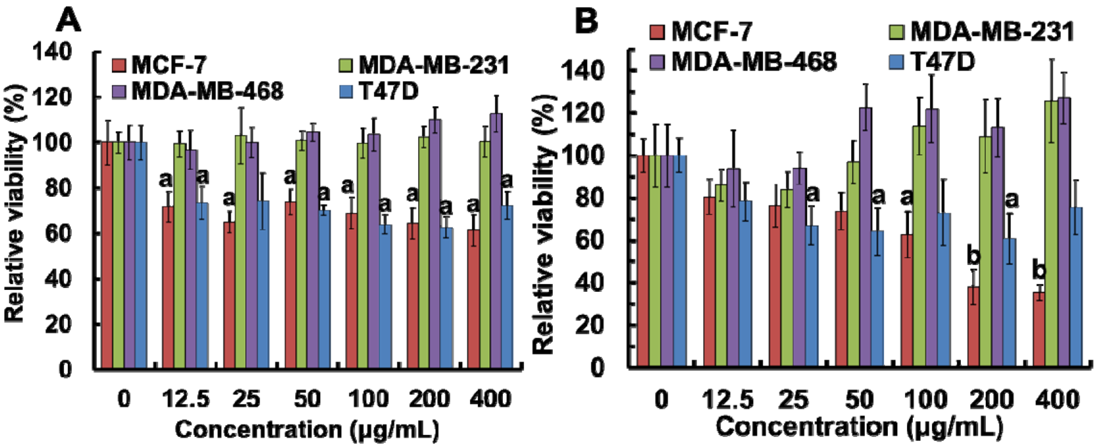

Supplementary Figure 1: Cell viability of different human breast cancer cells after treatment with LNT for 24 h **(A)** and 48 h **(B)** determined by MTT assay.

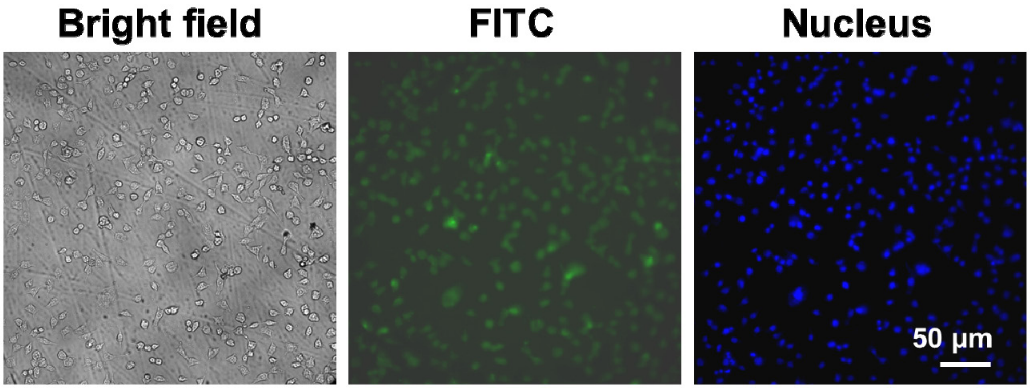

Supplementary Figure 2: Confocal images of MCF-7 cells with FITC after incubation for 40 min at 4°C. Cells were stained with Hoechst 33342, fixed and imaged using laser excitation wavelength at 405 and 488 nm. The micrographs were obtained at a magnification of 200 ×. Scale bar, 50 μm.

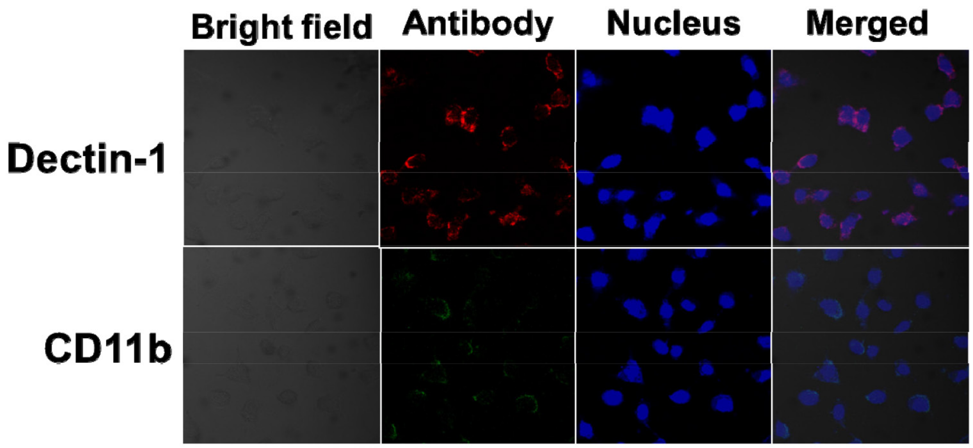

Supplementary Figure 3: Confocal images of MCF-7 cells after incubation with PE-anti-Dectin-1 (a) and FITC-anti-CD11b (b) antibody at 4°C for 40 min. Cells were stained with Hoechst blue, fixed and imaged using laser excitation at 405, 488 and 543 nm. The micrographs were obtained at a magnification of 200 ×.

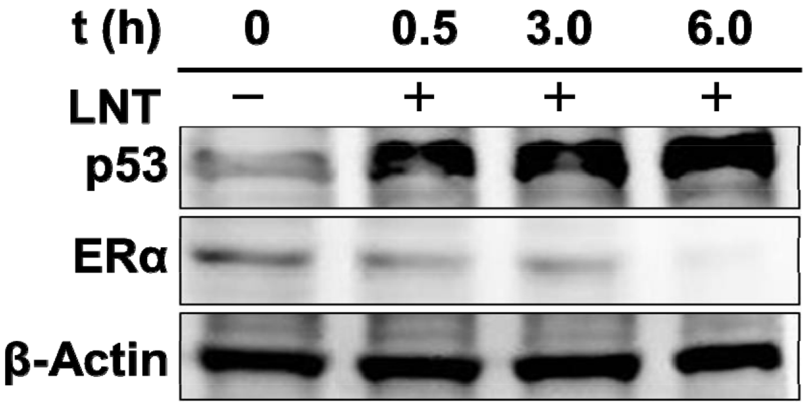

Supplementary Figure 4: p53 and ERα protein expression in T47D breast cancer cells detected by Western blotting analysis using their specific antibodies with β-Actin as the loading control. T47D cells were treated with 200 μ/mL LNT for indicated time intervals, and the whole cell extracts were prepared, followed by Western blotting analysis.

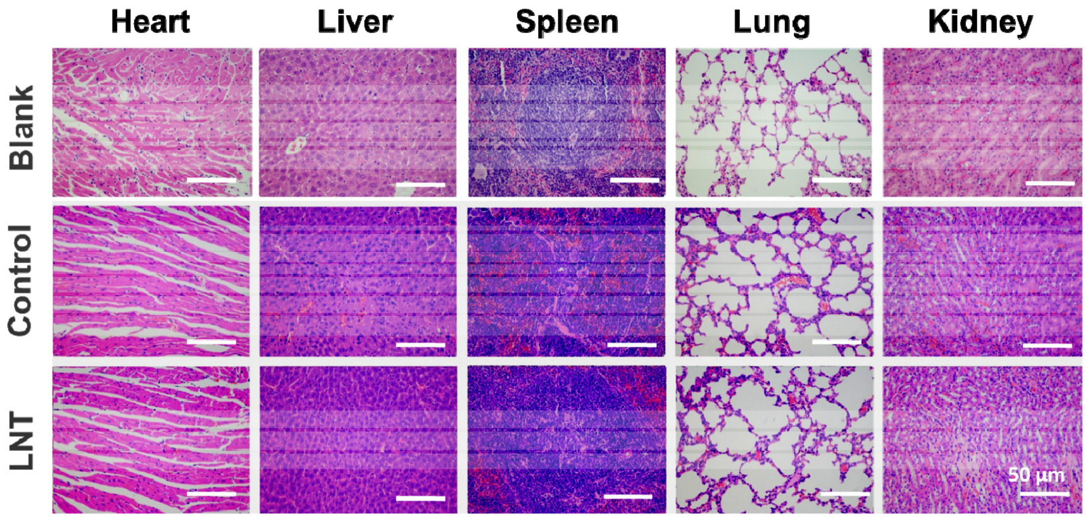

Supplementary Figure 5: Histologic assessments of major organs including heart, liver, spleen, lung and kidney with HE staining. The micrographs were obtained at a magnification of 200 ×. Scale bar, 50 µm.

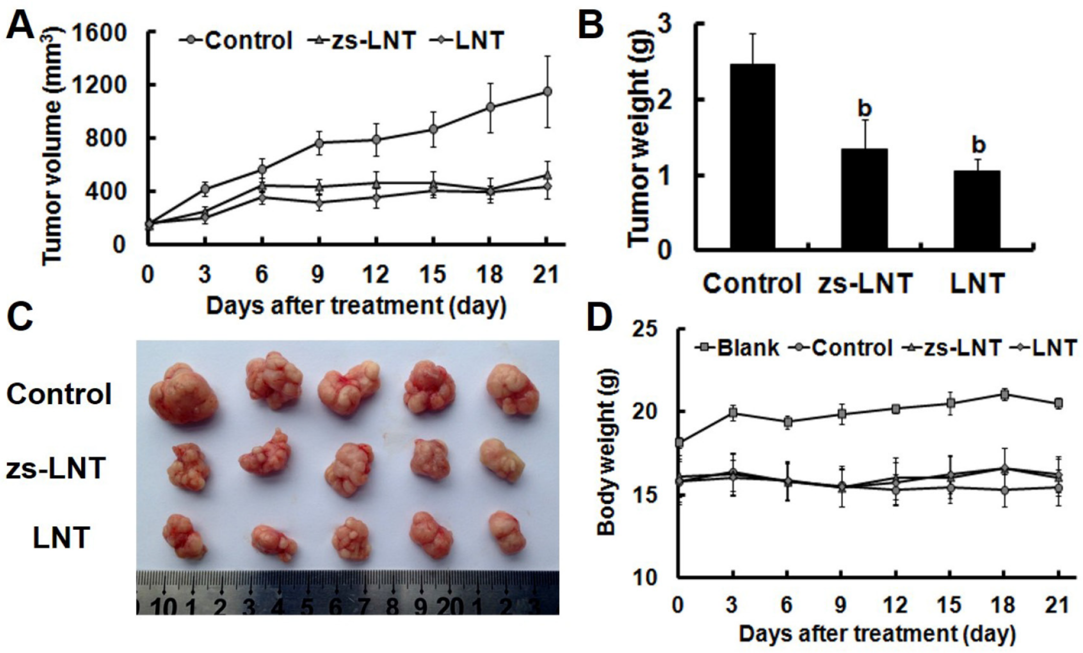

Supplementary Figure 6: Anti-tumor effects of LNT and zs-LNT against MCF-7 xenograft tumors *in vivo*. (A) Tumor sizes of the mice as a function of time. **(B)** Tumor weights at the end of the experiment. ^{b}p <0.001 versus control. The data *in vivo* are expressed as means ± SD of 5 mice in each group. **(C)** The photos of tumor tissue in different groups. **(D)** Body weights of mice as a function of treatment time.

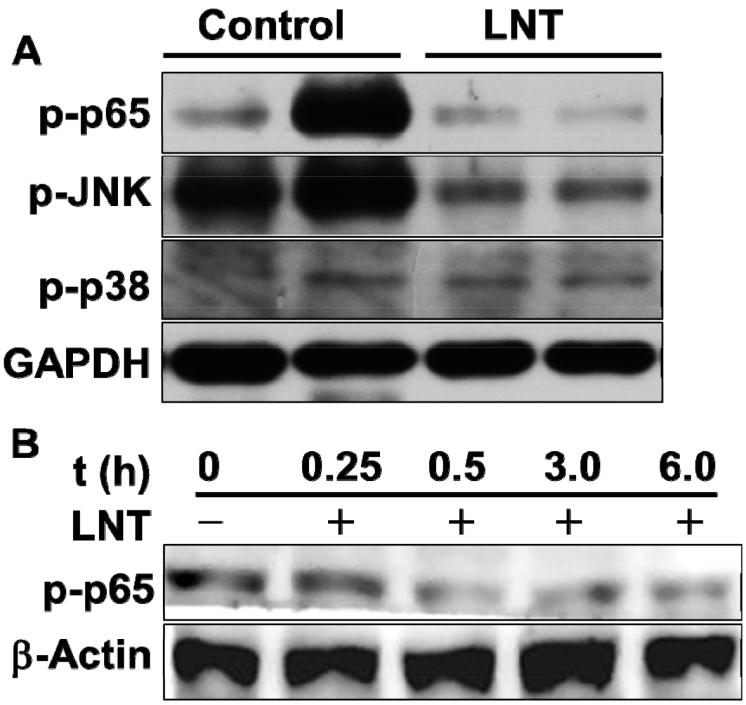

Supplementary Figure 7: Effects of LNT on NF-kB and MAPK activation in MCF-7 tumor tissues and cells. **(A)** LNT suppresses NF-KB p65 and JNK activation in MCF-7 tumor tissues detected by Western blotting analysis using their specific antibodies with GAPDH as the loading control. In each group, the proteins of tumor tissues from two mice were used. **(B)** LNT suppresses NF-KB p65 activation in MCF-7 cells. The whole proteins of MCF-7 cells after incubation with LNT (200 μg/mL) for different time were extracted, and were detected by Western blotting analysis using its specific antibody with β-Actin as the loading control. The results shown are representative of three independent experiments.

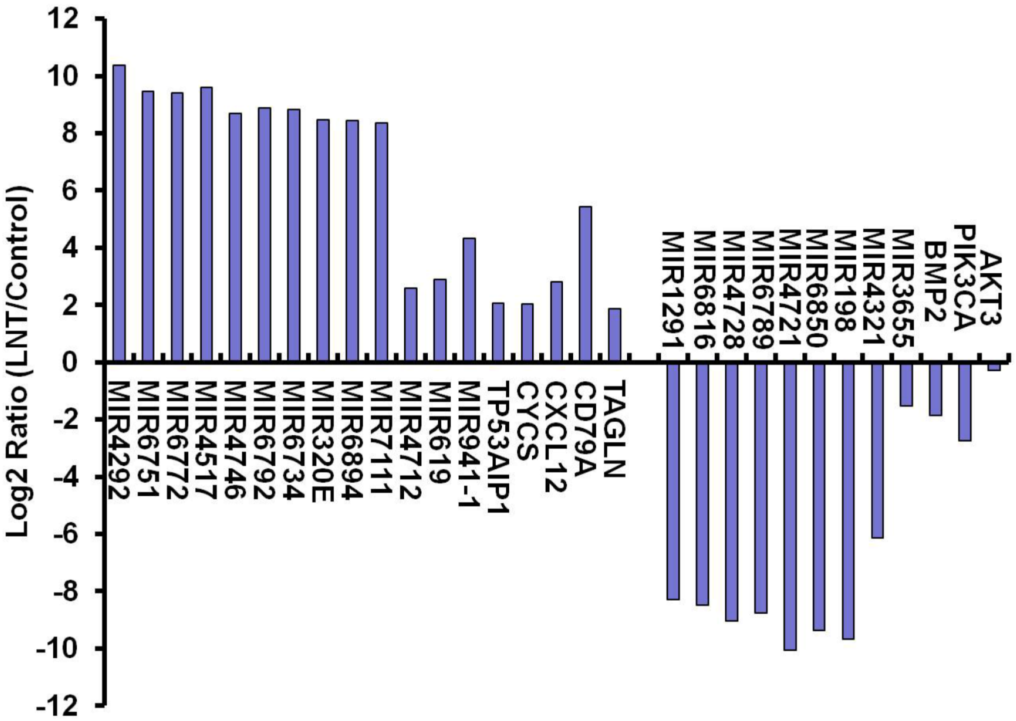

Supplementary Figure 8: Gene expression in tumor tissues. The fold change levels of some differentially expressed genes of corresponding heatmap.

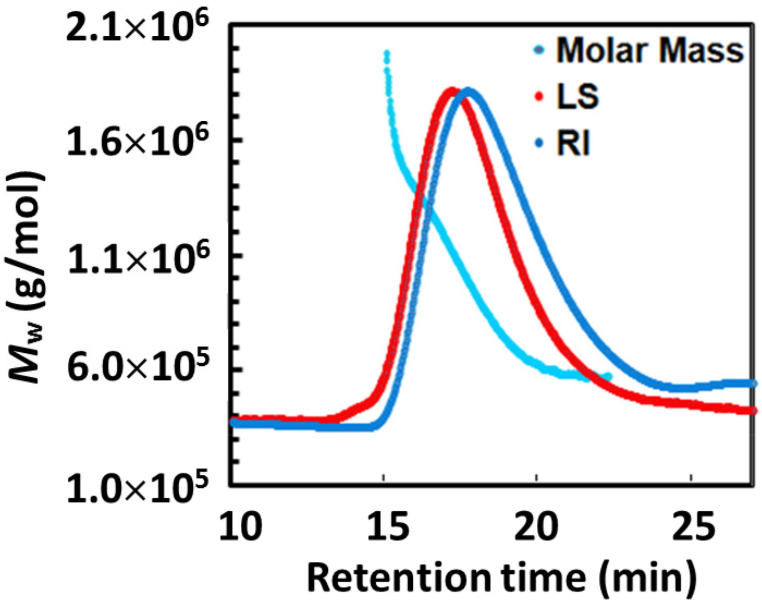

Supplementary Figure 9: SEC chromatogram and malor mass of the sample LNT in 0.9% $NaNO_3$ aqueous solution by SEC-MALLS at 25°C.
